# Pathogenesis of *Brucella abortus* and *Brucella melitensis* in bovine and ovine-derived trophoblasts and macrophages and impaired intracellular trafficking of the Rev1 vaccine strain

**DOI:** 10.1186/s13567-026-01781-3

**Published:** 2026-07-07

**Authors:** Aitor Elizalde-Bielsa, Maite Loperena-Barber, Christiane Pfarrer, Suzana P. Salcedo, Amaia Zúñiga-Ripa, Raquel Conde-Álvarez

**Affiliations:** 1 Departamento de Microbiología y Parasitología, Universidad de Navarra, and Instituto de Investigación Sanitaria de Navarra (IdiSNA), Navarra, Spain; 2 Unité de Recherche en Biologie des Microorganismes (URBM) and Namur Research Institute for Life Sciences (NARILIS), Department of Biology, University of Namur, Namur, Belgium; 3 Department of Anatomy, University of Veterinary Medicine Hannover, Bischofsholer Damm 15, Hannover, Germany; 4 Department of Pathobiological Sciences, University of Wisconsin-Madison, Madison, WI USA; 5 Laboratory of Molecular Microbiology and Structural Biochemistry, Centre National de La Recherche Scientifique UMR5086, Université de Lyon, Lyon, France

**Keywords:** *Brucella*, Rev1, intracellular trafficking, trophoblast, macrophage, AH-1, F3, BoMac, HeLa

## Abstract

*Brucella* spp. are Gram-negative facultative intracellular pathogens responsible for brucellosis, a widespread zoonosis. Among them, *B. melitensis* and *B. abortus* are the most epidemiologically relevant species, primarily infecting sheep and goats, and cattle, respectively. Upon infection, brucellae multiply within phagocytes and develop placental tropism, particularly targeting trophoblasts. Most studies of *Brucella* pathogenesis and vaccine development have relied on murine or human-derived epithelial and macrophagic cell lines, which may not fully reproduce the natural ruminant host. Here, we investigated whether the intracellular behaviour described for *Brucella* in non-natural-host models also occurs in ruminant trophoblasts (AH-1 and F3 cells) and macrophages (BoMac cells). Using confocal immunofluorescence microscopy and bacteriological assays, we observed similar infection rates for *B. abortus* and *B. melitensis*. By 24 hours post-infection (hpi), both species had transitioned to LAMP1-negative *Brucella*-containing vacuoles, with active replication occurring within calnexin-positive compartments. These results confirm that the *Brucella* intracellular trafficking previously described in non-natural-host models is conserved in these ruminant cells. Additionally, we characterised for the first time the intracellular behaviour of the Rev1 vaccine strain. Although its initial rate of infection was comparable to *B. melitensis* 16M, Rev1 exhibited a delay in LAMP1-exclusion from the *Brucella*-containing vacuole, with most bacteria remaining in LAMP1-positive compartments at 24 hpi, and showed delayed initiation of replication. These findings, together with slower in vitro growth, suggest that the Rev1 attenuation results from both delayed intracellular trafficking and impaired intracellular fitness. Our results advance the understanding of *Brucella* pathogenesis in natural-host and shed light on Rev1 attenuation, offering key insights to enhance current brucellosis vaccines.

**Supplementary Information:**

The online version contains supplementary material available at 10.1186/s13567-026-01781-3.

## Introduction

Brucellosis is a widespread zoonotic disease caused by bacteria of the genus *Brucella* [1]. This group of Gram-negative facultative intracellular coccobacilli includes a wide variety of nominal species with different preferential hosts, among them: *B. melitensis* (sheep and goats), *B. abortus* (cattle), *B. suis* (swine), *B. pinnipedialis* and *B. ceti* (marine mammals), *B. neotomae* (American woodrat), *B. microti* (European common vole), *B. canis* (dogs) and *B. ovis* (sheep) [2]. Beyond this diversity, two of the most epidemiologically relevant species are *B. melitensis* and *B. abortus*, whose control in livestock animals has been successfully achieved in some countries using the *B. melitensis* Rev1 and *B. abortus* S19 live-attenuated vaccines. However, these vaccines exhibit some limitations regarding their use in mass vaccination campaigns in endemic areas. None of these vaccines provides complete protection, vaccination of pregnant animals may induce abortions (especially in the case of Rev1) and, although of low virulence, these strains can infect humans with the added problem of the Rev1 being resistant to streptomycin, an antibiotic used to treat human brucellosis [3]. For these reasons, there is a pressing need to develop new vaccines or improve the currently available vaccines by solving their restraints while keeping their protective efficacy.

To achieve this objective, the evaluation of newly improved vaccine candidates ultimately relies on using *Brucella* natural hosts to determine the residual virulence, protection capacity and reproductive safety of the candidates. Although instrumental, the use of these animals for such assays is restricted due to their size, containment infrastructure, maintenance costs and ethical aspects. This way, outbred and inbred mice of several genetic backgrounds remain the main animal model for these aims [4, 5]. However, to reduce the number of animals required for these studies, biologically relevant cellular models may facilitate the assessment of the effect of mutations on *Brucella* fitness or for high-throughput screening of new vaccine candidates. Ex vivo placental explants and organoids preserve key aspects of placental architecture, allowing the study of dynamic materno-foetal cell interactions involved in colonisation and pathogenesis [6, 7]. Nevertheless, placental explants remain fully viable only for short periods in culture, which limits their usefulness for studying bacterial replication and trophoblastic responses throughout the course of infection. This short lifespan, together with the technical difficulty of cryopreserving placental explants, substantially reduces their practical applicability initial appeal [7, 8]. Placental organoids have been proposed as a model for studying the bovine placenta [9]; however, their three-dimensional structure restricts visual access to the organoid interior, complicating studies of intracellular trafficking of pathogens. For all these reasons, in vitro cell cultures remain a convenient and widely accessible model for studying intracellular pathogenic mechanisms.

The establishment of such cellular models requires an understanding of the intracellular life cycle of *Brucella* and, remarkably, this knowledge has been gained mainly using human HeLa epithelial cells and murine macrophages. In epithelial and macrophagic cells, infecting brucellae are internalised within a phagosome structure named the *Brucella*-containing vacuole (BCV). Within the first 8 hours (h) of infection, the endosomal BCV (eBCV) transits through an unaltered endocytic pathway acquiring early (e.g. Rab5 or EEA-1) and late (e.g. LAMP1/2 [lysosomal-associated membrane protein 1/2], CD63 or Rab7) endosomal markers [10–14]. These late endosomal markers control the fusion of the eBCV with late endocytic compartments and lysosomes, resulting in the acidification (pH 4.5–5) of the eBCV and its maturation towards a phagolysosome [13, 15, 16]. The acidification of the eBCV is a crucial step as it induces the expression of the Type 4 Secretion System (T4SS)-VirB, which begins to translocate effector molecules that modulate the biology of the host cell and mediate the interactions of the eBCV with ER- and Golgi apparatus-derived membranes. This way, between 8 and 12 hours post-infection (hpi), the eBCV progressively loses endosomal markers and acquires others associated with the membrane of the ER (e.g. calreticulin, calnexin or Sec61β) [10–12]. Hence, the eBCV converts into an ER-derived organelle [10–12, 17]. This phenomenon correlates with the beginning of bacterial replication within the vacuoles, which receive the name of replicative BCVs (rBCV) [18]. Finally, between the 48–72 hpi, the rBCVs are captured by autophagosome-like structures that originate multimembrane vacuoles, named autophagic BCV (aBCV), that acquire characteristics of late lysosomal and endosomal compartments (e.g. LAMP1, acidification) [18]. Complementary, although not fully understood, it has been shown that brucellae could also take advantage of other autophagy-related secretory mechanisms [19], such as the multivesicular body pathway [20] or mitophagy [21], to be liberated to the extracellular environment and continue to infect new cells, closing the cycle.

In order to develop biologically relevant cellular models, it is also essential to bear in mind the biology and pathogenesis of *Brucella.* These bacteria access the body via mucosal barriers and establish interactions with cells of the mononuclear phagocyte system, such as macrophages. Within these cells, brucellae avoid intracellular killing and persist to reach their preferential organs for replication [12, 22, 23], such as the spleen or the placenta, where brucellae replicate intracellularly at high numbers. In the case of the placenta, *Brucella* primarily targets trophoblasts and foetal-origin cells that establish the materno-foetal interface during pregnancy. Since trophoblast infection is a key step in the loss of placental integrity leading to abortion [24, 25], the use of trophoblastic cell lines may help to better understand the intracellular life/pathogenesis of *Brucella* and facilitate vaccine improvement by screening a large number of mutant and/or vaccine candidate strains. Thus, in this work, we evaluated the ability of *B. abortus* 2308W [26, 27] and *B. melitensis* 16M [28] to infect *Brucella* target cell lines (trophoblasts and macrophages) isolated from *Brucella* spp. hosts (sheep and cow) and we characterised the hallmarks of *Brucella* pathogenic intracellular trafficking within these cells: acquisition of LAMP1 (eBCV phase) and the subsequent loss of such marker and the acquisition of ER-associated calnexin (rBCV phase), and bacterial replication. Furthermore, we have analysed for the first time the intracellular basis of Rev1 vaccine attenuation in HeLa and ruminant target cell lines.

## Materials and methods

### Bacterial strains, cell lines and culture conditions

The different cell lines and bacterial strains used in this study are listed in Table 1 and Additional file 1, respectively. Bacteria were grown on TSB-D (Tryptic Soy Broth, bioMérieux), TSB with agar (European Bacteriological Agar, Condalab; TSA) or BAB2 with or without agar (Blood Agar Base No. 2, Oxoid) at 37 ºC and supplemented, when needed, with 50 µg/mL kanamycin and/or 12.5 µg/mL nalidixic acid. All strains were stored at –80 ºC in skim milk (Scharlau) or TYSB-DMSO (TSB supplemented with 0.5% yeast extract [Pronadisa, Conda], 5% foetal bovine serum [FBS, Sigma] and 7% dimethyl sulphoxide [VWR]).

**Table 1:** **Cell lines employed**

	**Characteristics**	**Source or reference**
**Cell lines**		
Trophoblasts		
AH-1	Ovine (*Ovis aries*) trophoblasts isolated from the cotyledon of a near-term pregnant Suffolk ewe and immortalised by transfection with a plasmid containing the SV40 large T antigen	[29]
F3	Bovine (*Bos taurus*) trophoblasts isolated from the cotyledon of a 5-month pregnant cow and spontaneously immortalised	[30, 31]
Macrophages		
BoMac	Bovine peritoneal macrophages isolated from a 13-year-old Jersey cow and immortalised by transfection with a plasmid containing the genomic DNA of SV40	[32]
Epithelial cells		
HeLa	Human epithelial cells derived from a cervical carcinoma from a 31-year-old patient	ATCC® CCL-2™

AH-1 ovine trophoblasts were grown in IMDM-GlutaMAX™ (Iscove’s Modified Dulbecco’s Medium containing GlutaMAX™ Supplement, Gibco) supplemented with 10% FBS. F3 bovine trophoblasts were expanded in DMEM/F12 (1:1) (Dulbecco’s Modified Eagle Medium/Nutrient Mixture F-12, Gibco) supplemented with 10% FBS. BoMac bovine peritoneal macrophages were grown in IMDM-GlutaMAX™ supplemented with 10% FBS, MEM NEAA (Minimum Essential Medium Non-Essential Amino Acids Solution, Gibco), MEM Vitamins (MEM Vitamin Solution, Gibco) and 50 µM 2-mercaptoethanol (Gibco). HeLa cells were cultured in DMEM (Gibco) supplemented with 10% FBS and 2 mM L-glutamine (Gibco). All cell lines were stored at −146 ºC in cryoprotectant media, i.e. the respective culture media supplemented with 50% FBS and 10% DMSO (VWR). Furthermore, all cell lines were routinely tested negative for *Mycoplasma* employing the LooKOut® *Mycoplasma* PCR Detection Kit (Sigma) or the Mycoalert™ *Mycoplasma* Detection Kit (Lonza).

### Generation of the GFP-expressing strains

GFP-expressing brucellae were obtained by stable insertion of the *gfpmut3* gene into the chromosome of the bacteria using the miniTn7 transposon [33]. Briefly, pUC18R6KT‐mini‐Tn7‐*gfp*‐Km, previously obtained in our laboratory [34], was transferred to the different *Brucella* strains by tetraparental conjugation with *E. coli* S17λpir (carrying pUC18R6KT‐mini‐Tn7‐*gfp*‐Km), SM10λpir (carrying pTNS2) and HB101 (carrying pRK2013). The exconjugants harbouring the inserted transposon were selected with 12.5 µg/mL nalidixic acid and 50 µg/mL kanamycin. The miniTn7 insertion was confirmed both by PCR with Glms_B/PTn7-R and PTn7-L/RecG-R specific primers to prove that the transposon was inserted immediately after *glmS* and before *recG* [33], and phenotypically checked by fluorescence microscopy.

### Cell infection

Cell lines were routinely cultured in their corresponding medium and maintained at 37ºC and a 5% CO_2_ atmosphere for one week prior to infection. Infections were performed as described elsewhere [35, 36]. Briefly, cells were seeded in 24 well-plates, containing 12 mm diameter glass coverslips (VWR) in the case of the infections for immunofluorescence (IF) microscopy, at the desired cell concentration: 1 × 10^4^ AH-1/well, 2 × 10^4^ F3/well, 2 × 10^4^ BoMac/well, and 2 × 10^4^ HeLa/well. On infection day, cells were counted, and the corresponding cell numbers were infected with a multiplicity of infection (MOI) of 1000 for HeLa and of 100 for AH-1, F3 and BoMac. After a centrifugation step at 400 × *g* for 10 min at 4 ºC, AH-1 and F3 trophoblasts were incubated for 30 min, BoMac macrophages for 15 min and HeLa cells for 1 h at 37 ºC with 5% CO_2_, to ensure a homogeneous bacterial internalisation. Then, to remove extracellular bacteria, cells were washed 3 times with fresh medium and incubated for 1 h with complete medium supplemented with 100 µg/mL of gentamicin. After that, the cells were maintained with media containing 25 µg/mL of gentamicin for the desired timespans. All experiments were performed in biological and/or technical triplicates and an attenuated *B. melitensis virB10*-mutant [11] was included as a control.

### CFU-counts

Cells were washed three times with DPBS (Dulbecco’s Phosphate Buffered Saline; Gibco) and incubated with 0.1% Triton X100 (Sigma) in DPBS for 5 min at room temperature. After the detergent treatment, the lysates were collected, tenfold diluted in DPBS, plated on BAB2 or TSA, and incubated at 37 ºC for approximately 4–5 days to determine the number of intracellular bacteria.

### Fluorescence microscopy

At the desired time points, coverslips were washed and fixed with AntigenFix (Diapath) at 37 ºC for 20 min. Immunostaining was performed following the previously described methodology [37].

For LAMP1 immunostaining, coverslips were directly incubated for 1 h at room temperature with the primary anti-LAMP1 antibody (rabbit polyclonal; Abbott, ab24170) in a PBS (Gibco) solution containing 0.2% saponin and 10% horse serum (Gibco). After washing the coverslips with a 0.1% saponin solution in PBS, samples were incubated for 1 h at room temperature with the secondary antibody Alexa Fluor™ 555 donkey anti-rabbit IgG (H + L) (Invitrogen, A6572) and DAPI (Invitrogen) in PBS containing 0.2% saponin and 10% horse serum.

For ER immunostaining, permeabilisation was performed by incubation in a PBS solution containing 0.1% Tween® 20 (Euromedex), 1% BSA (Bovine Serum Albumin Standard; Euromedex), 10% horse serum and 0.3 M Glycine (Euromedex) for 5 min at room temperature. After a wash with PBS, coverslips were blocked in a PBS solution containing 0.5% saponin (Sigma, 47,036) and 1% BSA for 5 min at room temperature. Then, cells were incubated overnight at 4 ºC with the primary anti-calnexin antibody (rabbit polyclonal; Abbot, ab22595) in a 1% BSA-PBS solution. Subsequently, the coverslips were washed and incubated for 1 h at room temperature with the same secondary antibody used in the LAMP1-staining and DAPI in a 1% BSA-PBS solution.

Finally, the stained coverslips were washed and mounted on a glass slide with ProLong™ Gold antifade reagent (Invitrogen Technologies). Coverslips were visualised with a Confocal Zeiss inverted laser-scanning microscope LSM800 and processed using FigureJ and ImageJ software.

### Statistical analysis

Statistical comparisons for the infectivity and IF-quantification of BCV LAMP1-kinetics experiments were made by ordinary one-way ANOVA and Tukey’s or Sidak’s multiple comparisons post-hoc test. Comparisons between the bacterial replication levels were done by unpaired t-test and comparisons between categories of intracellular bacterial burden were made using two-way ANOVA and Sidak’s correction.

## Results

### AH-1 ovine trophoblasts, F3 bovine trophoblasts, and BoMac bovine macrophages are permissive to *B. abortus *and *B. melitensis* infection

First, to study the intracellular trafficking of *B. abortus* and *B. melitensis*, we constructed GFP-expressing *B. abortus* 2308W (*Ba*2308W) [26, 27] and *B. melitensis* 16M (*Bm*16M) [28] wild-type and *Bm*16MΔ*virB* mutant strains by stable insertion of the *gfpmut3* gene in the corresponding genomes employing the miniTn7 transposon [33]. Then, to confirm that the miniTn7 insertion and the *gfpmut3* gene expression did not affect bacterial growth, we analysed the growth curves of both the GFP-expressing strains and their respective parental strains in rich media and observed no remarkable growth differences between fluorescent and non-fluorescent strains (Additional file 2).

**Figure 1:**
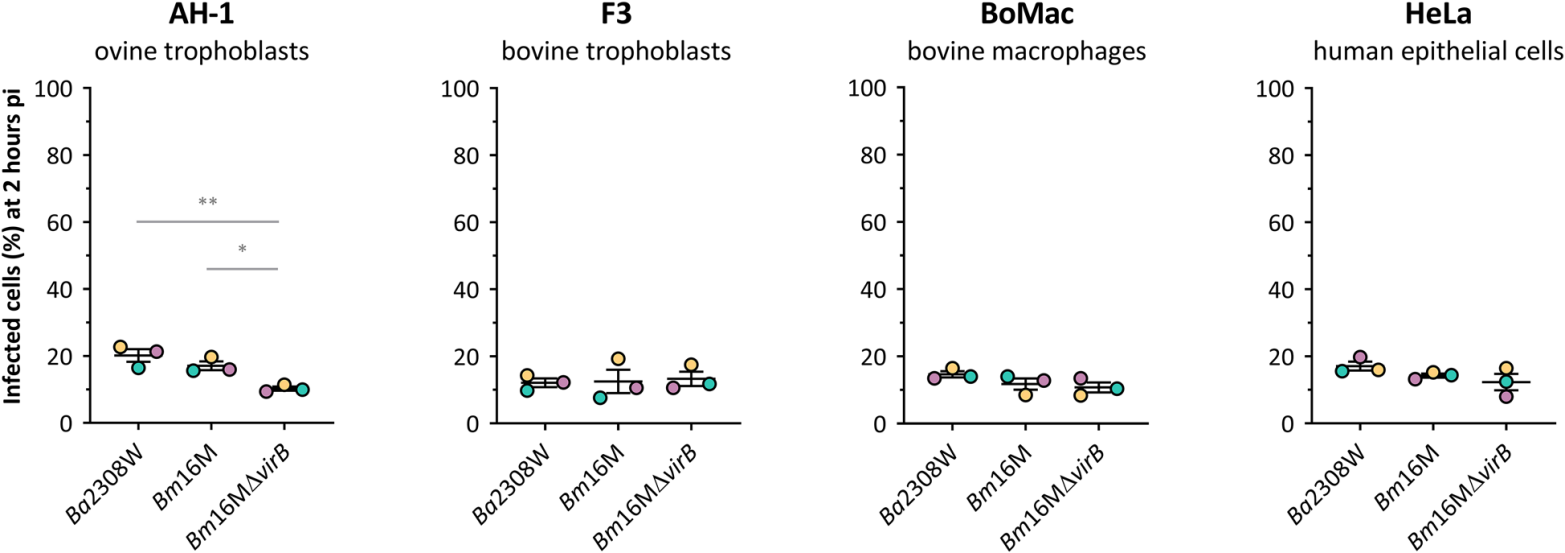
**Infection rates of *B. abortus*** **(*Ba*2308W) and *B. melitensis*** **(*Bm*16M) were similar regardless of the cell line.** Infectivity was determined as the number of infected cells at 2 hpi from 20 screened quadrants (minimum of 140 cells screened) and expressed as mean percentage of infected cells ± SEM, obtained from independent biological triplicates (colour coded: yellow, blue, purple). Statistical comparisons were made by ordinary one-way ANOVA and Tukey’s multiple comparisons post-hoc test (* *p* < 0.05; ** *p* < 0.01).

Once validated, we carried out cell infections with these GFP-expressing strains at an MOI of 100 (AH-1, F3 and BoMac) or 1000 in the case of the HeLa cells, the latter being used as a control of a well-established cellular model in *Brucella* research. We first studied the entry (2 hpi) of the different strains in the four cell lines by confocal IF microscopy. We observed that *Ba*2308W and *Bm*16M showed similar infection rates in the three ruminant cell lines with values ranging 12–18% of infected cells. The infection rates reached in trophoblasts and macrophages were similar to the ones obtained in HeLa cells, but the MOI employed in the epithelial cells was 10 times greater. This fact is not surprising for BoMac, a macrophagic cell line, but in the case of the trophoblastic cell lines (AH-1 and F3), it might reflect the phagocytic competence of this cell type. Regarding the *Bm*16MΔ*virB* control, we observed similar infection rates to those of *Ba*2308W and *Bm*16M in F3, BoMac and HeLa cells at 2 hpi while, in AH-1 cells, the *virB*-mutant showed a lower infection rate (Figure 1).

### ***B. abortus*** and ***B. melitensis*** traffic in a transitory LAMP1^+^ compartment and establish a calnexin^+^ replicative niche in the AH-1, F3 and BoMac ruminant cells

**Figure 2:**
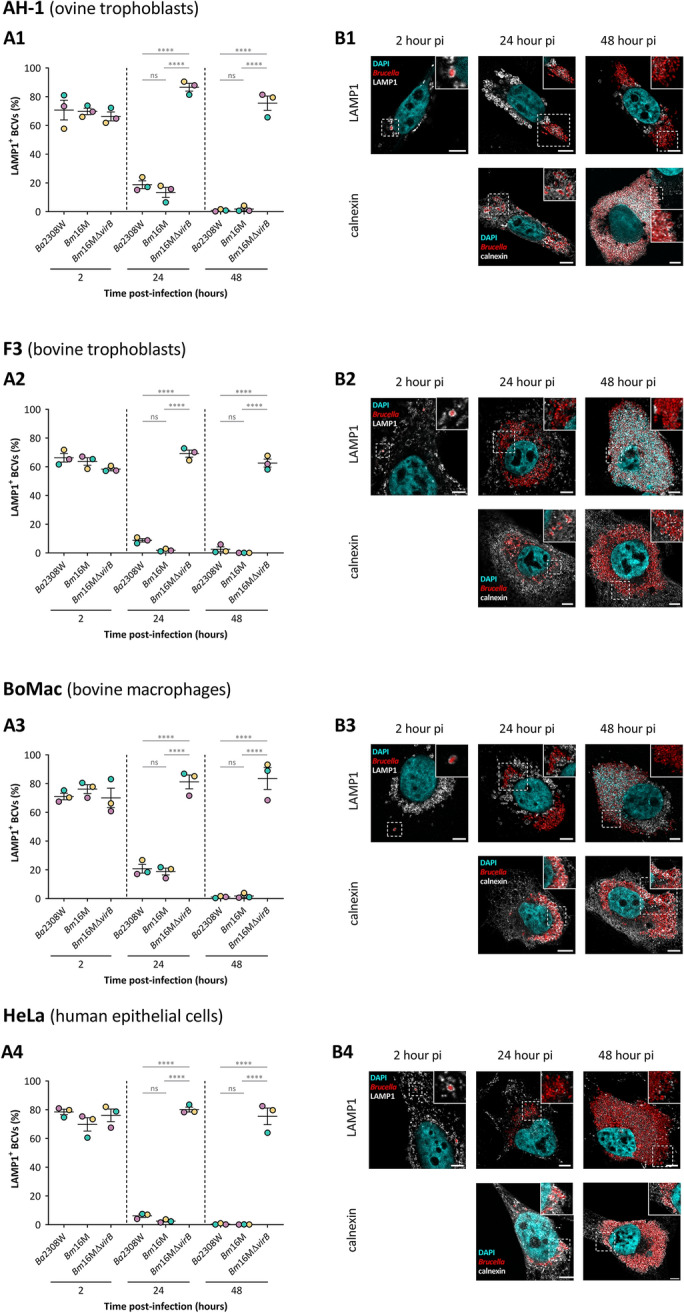
***B. *abortus (*Ba*2308W) and *B. melitensis* (*Bm*16M) traffic within LAMP1**^**+**^** BCVs that mature to LAMP1**^**−**^**/calnexin**^**+**^** compartments**. **A** LAMP1 positivity was determined for each of the BCVs found within each 50 infected cell and expressed as mean percentage of LAMP1^+^-BCVs/infected-cell ± SEM, obtained from independent biological triplicates (colour coded: yellow, blue, purple). Statistical comparisons were made by ordinary one-way ANOVA and Sidak’s multiple comparisons post-hoc test (* *p* < 0.05; ** *p* < 0.01; *** *p* < 0.001; **** *p* < 0.0001). **B** Representative pictures of wild-type *B. abortus* or *B. melitensis* LAMP1^+^ BCVs with internalised brucellae at 2 hpi and LAMP1^−^/calnexin^+^ BCVs with replicating bacteria at 24 and 48 hpi. DAPI: cyan channel; brucellae: red channel; LAMP1/calnexin: white channel. Scale bars correspond to 5 µm.

We next characterised the LAMP1 kinetics of BCVs by confocal IF microscopy, as in most established cellular models, brucellae first traffic within LAMP1^+^ BCVs before establishing a replicative niche in a LAMP1^−^/calnexin^+^ compartment. We found similar BCVs LAMP1-kinetic profiles in the three ruminant cell lines as in HeLa cells. At 2 hpi, *Ba*2308W and *Bm*16M were contained in 60–80% LAMP1^+^ BCVs in the four cell lines (Figure 2, panels A1-4). Then, at 24 hpi, both *Ba*2308W- and *Bm*16M-containing BCVs lost LAMP1, with values below 20%, and began to significantly replicate (see below) in a compartment that started to label with the ER marker calnexin (Figure 2, panels B1-4). Accordingly, LAMP1 presence on BCVs was near-zero in the four cell lines for both strains at 48 hpi (Figure 2, panels A1-4), when both *Ba*2308W and *Bm*16M multiplied in calnexin-positive BCVs (Figure 2, panels B1-4; see below). Consistent with the previously reported incapability of VirB-mutants to escape the phagolysosomal pathway and reach the calnexin^+^ replicative compartment, and in accordance with the results in HeLa cells, *Bm*16MΔ*virB* retained the LAMP1^+^ marker on BCVs over the 48 h of infection in the AH-1, F3 and BoMac cell lines (Figure 2, panels A1-4).

### ***B. abortus*** and ***B. melitensis*** replicate in high numbers within the LAMP1^−^/calnexin^+^ replicative niche in the AH-1 ovine trophoblasts, F3 bovine trophoblasts, and BoMac bovine macrophages

**Figure 3:**
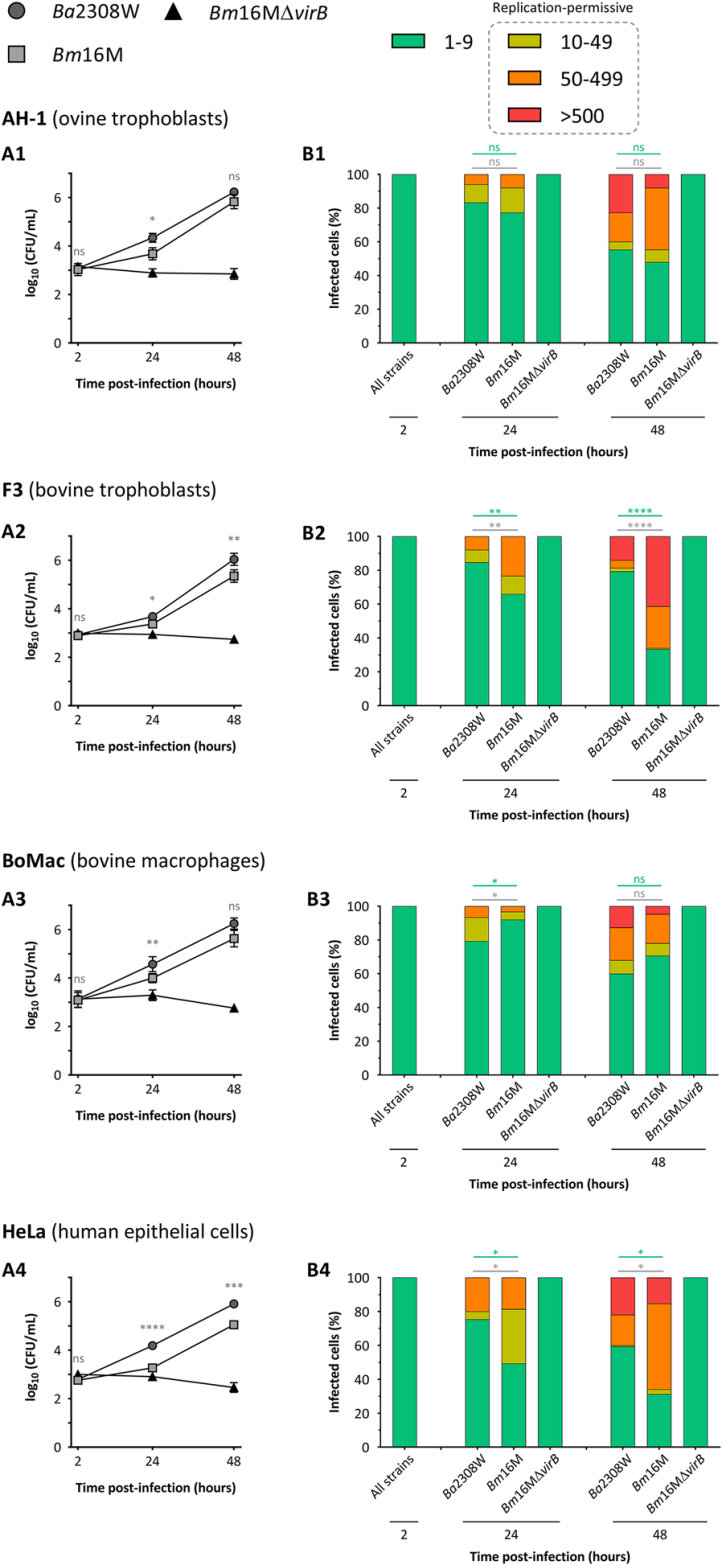
***B. abortus*** **(*Ba*2308W) replicates in higher total numbers within infected cells and** ***B.*** **melitensis (*Bm*16M) induces a higher number of replication-permissive infected cells.** **A** Bacterial replication levels were determined by CFU-counting and values were expressed as mean log_10_(CFU/mL) ± SD; obtained from technical triplicates and performed two times obtaining similar results. **B** Bacterial replication profiles were determined by IF microscopy by estimation of intracellular bacterial burden based on the surface of fluorescent signal along the Z-stack within 50-infected-cells, and values were classified within the categories of non-replicative (0–9), initiating low replication (10–49), high replication (50–499) and extensive replication (> 500 intracellular bacteria); obtained from biological triplicates. Statistical comparisons between *Ba*2308W and *Bm*16M were made by unpaired t-test (A) or by two-way ANOVA and Sidak’s correction (B) (* *p* < 0.05; ** *p* < 0.01; *** *p* < 0.001; **** *p* < 0.0001).

We next studied the replication kinetics (i.e. intracellular bacterial numbers) of *Ba*2308W and *Bm*16M in the four cell lines by CFU-counting. Both strains multiplied extensively in the four cell lines, replicating already between the 2- and 24-h time-points, with *Ba*2308W showing a greater increment in bacterial burden, regardless of the cell type and origin (Figure 3, panels A1-4). Then, between the 24- and 48-hpi, both strains experienced a similar increment in intracellular bacteria, leading to higher intracellular bacterial numbers for the *Ba*2308W strain, particularly noticeable in HeLa cells. As expected from the LAMP1 kinetics results, *Bm*16MΔ*virB* was unable to replicate in any cell line (Figure 3, panels A1-4).

Finally, we complemented the CFU replication kinetics by studying the profiles of intracellular bacterial replication (i.e. the intracellular bacterial burden within each individual infected cell) by confocal IF microscopy and confirmed the ability of *Ba*2308W and *Bm*16M to multiply in the ruminant cell lines and reach high bacterial numbers per cell (Figure 3, panels B1-4; Additional file 3). At 2 hpi, the infected cells contained less than 10 intracellular bacteria regardless of the strain. As infection time progressed, *B. abortus* and *B. melitensis* replicated in some infected cells, showing an increase in the intracellular bacterial burdens, with a predominance of low (10–49 intracellular bacteria) and high (50–499 intracellular bacteria) replication-permissive cells at 24 hpi and then, progressing to a predominance of high and extensive (>500 intracellular bacteria) replication-permissive cells at 48 hpi (Figure 3, panels B1-4; Additional file 3). Interestingly, *Bm*16M induced a higher proportion of replication-permissive cells (i.e. in the > 10 intracellular bacteria/infected-cell categories) in bovine trophoblasts and HeLa cells. This was not the case for BoMac macrophages, where a statistically higher proportion of replication-permissive cells was observed for *Ba*2308W at 24 h, although not significant at 48 hpi. Consistent with the LAMP1 kinetics and the total bacterial replication values, *Bm*16MΔ*virB*-infected cells showed no increment of intracellular bacterial burdens with an absence of replication permissive cells (Figure 3, panels B1-4; Additional file 3).

In conclusion, *Ba*2308W and *Bm*16M wild-type strains were able to multiply in the ruminant AH-1 and F3 trophoblast and BoMac macrophage cell lines, validating their use as biologically relevant models for the study of *Brucella* intracellular pathogenesis.

### The delayed intracellular trafficking of the Rev1 vaccine strain contributes to its intracellular attenuation

Rev1 is a live attenuated vaccine that is the only brucellosis vaccine recommended by the World Organization for Animal Health for small ruminants [3, 38]. Although several works have underpinned the differences found in this strain in relation to the *B. melitensis* 16M reference strain or other *B. melitensis* strains at the genetic or proteomic level, a comprehensive mechanism for the attenuation of the Rev1 vaccine has not been clearly described yet [39–41]. Therefore, we sought to assess how this attenuation occurs at the intracellular level to set up the basis for the study and improvement of this vaccine. Hence, once we had characterised *B. melitensis* and *B. abortus* intracellular behaviour in the three ruminant cell lines, we extended this research to the Rev1 vaccine. For this, we also generated a GFP-expressing Rev1 strain using the pUC18R6KT‐mini‐Tn7‐*gfp*‐Km plasmid and confirmed that GFP expression did not affect bacterial growth (Additional file 2). Interestingly, we also noted that the Rev1 vaccine strain exhibited a growth delay in rich media, with significantly longer generation times than *Bm*16M, particularly in TSB medium (33.6 h for Rev1 versus 7.9 h for *Bm*16M). Additionally, the Rev1 strain produced a lower total biomass in these conditions, as reflected by the maximum OD values reached (0.72 for Rev1 compared to 1.06 for *Bm*16M).

**Figure 4:**
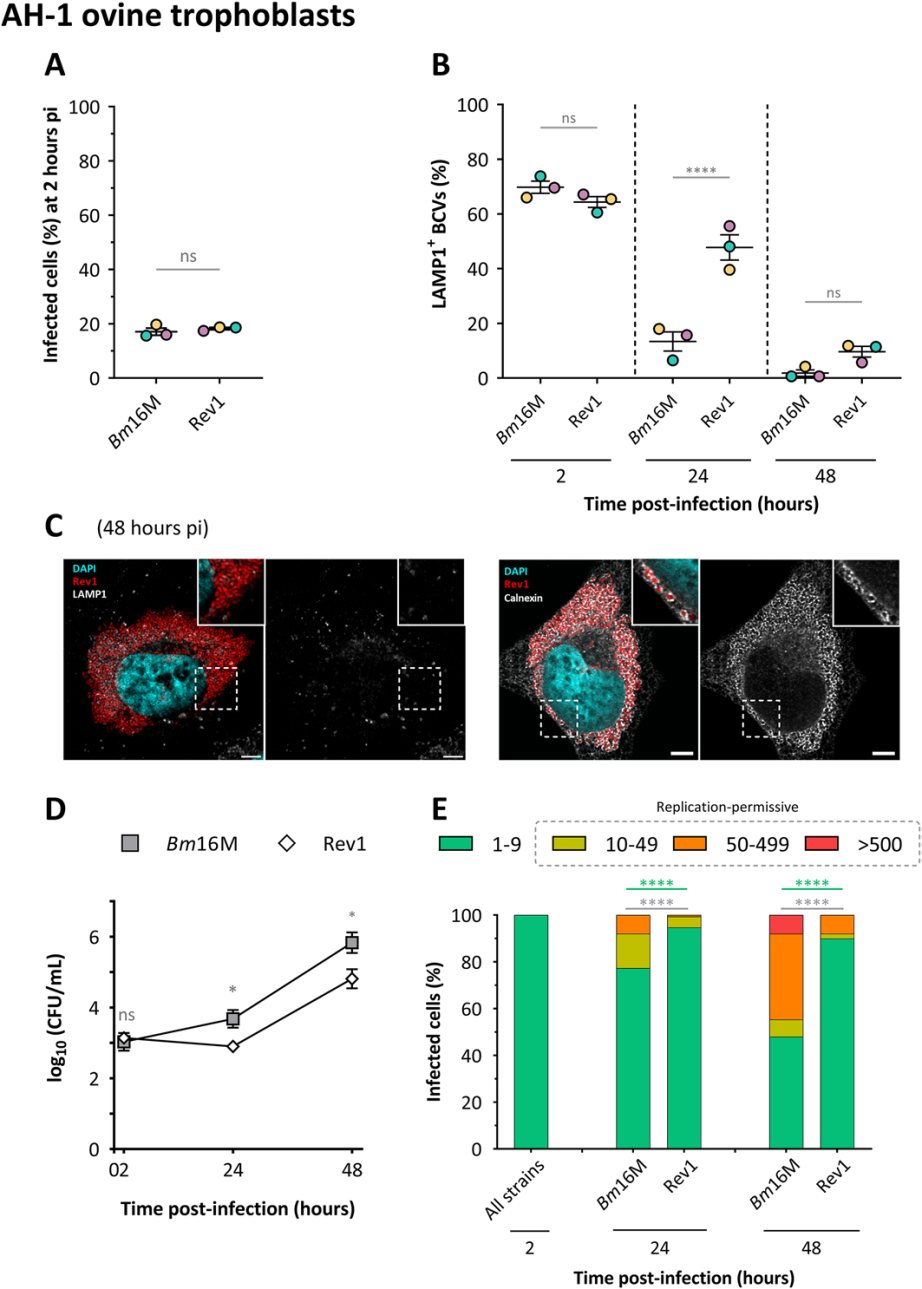
**The Rev1 attenuated vaccine-strain infects AH-1 ovine trophoblasts at the same level as the *B.*** **melitensis (*Bm*16M) parental strain but develops an imperfect intracellular trafficking and a delayed intracellular multiplication in LAMP1**^**−**^**/calnexin**^**+**^ **BCVs with lower replication values.** **A** Infectivity was determined as the number of infected cells at 2 hpi from 20 screened quadrants (minimum of 140 cells screened) and expressed as mean percentage of infected cells ± SEM, obtained from independent biological triplicates (colour coded: yellow, blue, purple). **B** LAMP1 positivity was determined for each of the BCVs found within each 50 infected cell and expressed as mean percentage of LAMP1^+^-BCVs/infected-cell ± SEM, obtained from independent biological triplicates (colour coded: yellow, blue, purple). **C** Representative pictures of Rev1-infected AH-1 ovine trophoblasts at 48 hpi with LAMP1^+^ BCVs (left panel) and calnexin^+^ BCVs (right panel) with replicating bacteria. (DAPI: cyan channel; Brucellae: red channel; LAMP1/calnexin: white channel. Scale bars correspond to 5 µm). **D** Bacterial replication levels were determined by CFU-counting and values were expressed as mean log_10_(CFU/mL) ± SD; obtained from technical triplicates and performed two times obtaining similar results. **E** Bacterial replication profiles were determined by IF microscopy by estimation of intracellular bacterial burden based on the surface of fluorescent signal along the Z-stack within 50 infected cells and values were classified within the categories of non-replicative (0–9), initiating low replication (10–49), high replication (50–499) and extensive replication (> 500 intracellular bacteria); obtained from biological triplicates. Statistical comparisons were made by ordinary one-way ANOVA and Tukey’s (A) or Sidak’s (B) multiple comparisons post-hoc tests, by unpaired t-test (**D**) or by two-way ANOVA and Sidak’s correction (D): *ns* = not significant; * *p* < 0.05; ** *p* < 0.01; *** *p* < 0.001; **** *p* < 0.0001. Results for the *Bm*16M strain are duplicated from the ones found in Figures. 1, 2 and 3.

For the Rev1 intracellular trafficking assays, since the results were similar in the four cell lines, we present the results regarding the AH-1 ovine trophoblasts in Figure 4 and the ones regarding the bovine F3 trophoblasts and BoMac macrophages and the HeLa cells in Additional file 4. First, regarding bacterial entry, we found no differences between the number of infected cells with Rev1 and *Bm*16M at 2 hpi in any of the cell lines (Figure 4, panel A; Additional file 4), indicating that Rev1 attenuation is not related to cell entry. Then, we observed that Rev1 BCVs took longer to lose LAMP1 in the different cell lines (Figure 4, panel B), suggesting a delay in escaping the phagolysosomal pathway. At 24 hpi, the percentage of LAMP1^+^ Rev1 BCVs was higher than that of the parental *Bm*16M but reduced compared to the *Bm*16MΔ*virB* mutant (Figure 4, panel B). Nonetheless, at 48 hpi less than 20% of the Rev1 BCVs maintained the LAMP1-labeling in the four cell lines, which was concomitant with calnexin recruitment and Rev1 replication (Figure 4, panels B and C; Additional file 4). Hence, these results indicate that Rev1 exhibits an initial intracellular trafficking delay, although that does not prevent the vaccine strain from reaching the LAMP1^−^/calnexin^+^ replicative compartment and multiplying within infected cells.

Regarding replication, CFU-counting experiments showed intracellular bacterial numbers significantly reduced when compared to *Bm*16M (Figure 4, panel D) in the four cell lines. At 24 hpi, some Rev1-infected cells began to show signs of replication permissiveness with the initiation of bacterial intracellular replication in calnexin^+^ BCVs but significantly reduced when compared to *Bm*16M intracellular burdens (Figure 4, panel E). This is consistent with the lack of increment of Rev1 intracellular bacterial numbers between the 2 and 24 hpi (Figure 4, panel D). The reduced levels of Rev1 intracellular replication agreed with the growth delay observed in culture (Additional file 2) and with more than 95% of cells in the non-permissive category (1–9 bacteria/cell) for all the cell lines (Figure 4, panel E; Additional file 3). Rev1 started to replicate between the 24 and 48 h time points, showing a significant increase in the total bacterial numbers in all the cell lines (Figure 4, panel D) but with a lower replication rate than *Bm*16M (AH-1: 89.3 ± 38.2 and 144.0 ± 63.3; F3: 24.5 ± 8.0 and 106.0 ± 31.4; BoMac: 6.4 ± 3.0 and 47.1 ± 19.7; HeLa: 17.8 ± 6.1 and 59.6 ± 9.5 for Rev1 and *Bm*16M, respectively; Additional file 5). This increase in intracellular burden was evidenced as an increase of replication permissive infected cells bearing 50–499 intracellular bacteria but did not reach > 500 bacteria/cell (Figure 4, panel E).

Thus, although Rev1 did not reach intracellular replication levels comparable to those of *Bm*16M in any cell line at the studied time points, the vaccine strain was able to replicate in considerably high numbers in the tested cell lines.

## Discussion

The majority of the insights into the basis of *Brucella* intracellular life cycle have been historically elucidated using human HeLa epithelial cells and murine macrophages. These widely employed cellular models have been of undoubted value for this purpose, but the host preference exerted by the different *Brucella* spp. may underpin the interest in using cell cultures obtained from these ruminant hosts as more biologically relevant models for such an animal pathogen, in the same way as it has been done for other zoonoses of global relevance (see below), or to some extent also explored at the cytokine profile with *B. canis* on primary canine trophoblasts [42]. Such interest was already pointed out by García-Méndez et al., who stated that discrepancies in differential *Brucella* infection outcome “*could be attributed to (…) Brucella species‐dependent response of trophoblasts*” [43]. With these ideas in mind, we worked with trophoblast and macrophage cell lines obtained from *Brucella* natural hosts: ovine AH-1 trophoblasts [29], bovine F3 trophoblasts [30, 31] and BoMac peritoneal macrophages [32], extensively used to study the pathobiology of other intracellular and/or abortifacient pathogens, but not yet of *Brucella* spp. The AH-1 ovine trophoblasts have been used to study the obligate intracellular protozoa *Neospora caninum* [29] and *Toxoplasma gondii* [44], or the obligate intracellular bacteria *Chlamydia abortus* [45] and *Waddlia chondrophila* [46], all of them able to infect and replicate within placental trophoblasts leading to pregnancy failure in small ruminants or cattle [47–49]. The F3 bovine trophoblasts have also been used to study *N. caninum* [50], as well as *Coxiella burnetii*, an obligate intracellular bacterium that infects and replicates within trophoblasts resulting in reproductive failure in cattle [51]. Regarding the BoMac cell line, these bovine macrophages have been extensively used to study intracellular bacteria such as mycobacteria [52], *Mycoplasma bovis* [53], *Listeria monocytogenes* [54], different *Salmonella* species [55] or the intracellular protozoa *Theileria annulata* [56] and *Plasmodium falciparum* [57]. In addition, we recently used this bovine macrophagic cell line to characterise the potential virulence of a *Pseudochrobactrum* species newly isolated from cows [58].

*B. abortus* and *B. melitensis* displayed similar intracellular trafficking and replication kinetics to the ones previously described in classical in vitro models, such as HeLa cells, also used in this study, that were dependent on a functional T4SS-VirB system. Although HeLa epithelial cells are widely used to study *Brucella* intracellular pathogenesis, brucellae do not efficiently invade these cells and thus very high infectious doses are used in most studies. Note that, for example, MOI values of 1–10 or 10–50 are typically used for HeLa cells in *Chlamydia* [59–61] or *Coxiella* [62–64] studies, respectively. In the ruminant-derived cell lines used in this study, we obtained a rate of infection similar to that of HeLa cells with an MOI ten times lower. This may be explained by the phagocytic ability of trophoblasts in vivo [65], as they actively participate in uterus remodelling throughout pregnancy [66, 67] and can phagocytose maternal erythrocytes at the placenta [68]; or by the specific targeting of this cell type by *Brucella* [25, 69, 70], as the ability to reach, infect and/or replicate within trophoblasts is not a widely distributed feature among pathogens [71–73]. This aspect raises the question of how efficient or unique this *Brucella* reproductive-targeting and ability to develop pathology and replicate within trophoblasts may be compared to other intracellular pathogens with no reproductive tropism.

Notably, in the microscopy studies we observed that, in both HeLa cells and bovine trophoblasts, *Bm*16M replicated within a higher proportion of replication-permissive cells, whereas *Ba*2308W yielded higher total bacterial counts in CFU assays. However, this pattern was less pronounced in AH-1 trophoblastic cells. These findings suggest that the intracellular behaviour of the two species is distinct, with differences in the frequency of replication-permissive cells and in the bacterial loads achieved within them. It is tempting to speculate that such differential behaviours may reflect distinct intracellular replication strategies, potentially linked to the different genomic contexts of *B. melitensis* and *B. abortus* [74]. However, the variation of this phenotype across cell lines indicates that the outcome of infection does not solely depend on intrinsic bacterial traits, but is also shaped by the cell line characteristics. Consistently, interspecies differences were previously reported in JEG-3 human trophoblasts. In these extravillous trophoblasts (EVTs), *B. melitensis* reached the canonical replicative niche, whereas *B. abortus* replicated within a LAMP1⁺/calnexin⁻ atypical compartment, a distinction that was not observed in BeWo cytotrophoblasts [35]. It would be interesting to verify if such differences in the replicative niche nature extend to other EVT-like cell lines, like the Swan-71 or the HPT-8 human trophoblasts, within which the CFU replication levels, but not the replicative compartment nature, have already been explored for *B. abortus* and *B. melitensis* wild-type and several mutant strains [75–77]. EVTs play a central role in decidualisation and remodelling of the maternal portion of the haemochorial placenta and are characterised by pronounced invasive and phagocytic activities [65]. In particular, endovascular EVTs establish direct contact with maternal blood and have been shown to phagocytose erythrocytes. Such heightened phagocytic activity may influence intracellular trafficking dynamics and potentially hinder access to the classical replicative niche, as suggested for *B. abortus* in JEG-3 cells [78]. In contrast to human trophoblastic cell lines, ruminant trophoblast models remain comparatively underused and functionally less defined. This limited characterisation hinders the interpretation of species-dependent intracellular behaviours in these models. As trophoblastic cell lines may differ in differentiation status, endocytic capacity and vesicular dynamics, among others, functional heterogeneity among ruminant trophoblastic models may modulate the intracellular fate of *B. melitensis* and *B. abortus*. In this context, AH-1 and F3 cells may represent distinct trophoblastic states, potentially contributing, together with intrinsic bacterial differences, to the differential behaviours observed in our study. Moreover, AH-1 and F3 cells may differentially reveal phenotypes associated with specific virulence factor mutations, suggesting that both cell lines could serve as complementary models for studying *Brucella* spp. intracellular behaviour.

Regarding the Rev1 vaccine intracellular behaviour, as we previously mentioned, this vaccine is a spontaneously attenuated strain [79] whose basis of attenuation is not fully understood. In this work, we have shown that the Rev1 vaccine strain displays a delayed ability to lose LAMP1 at the BCV, indicating difficulty in escaping the phagolysosomal pathway and inducing the formation of the rBCV. This delayed intracellular trafficking of Rev1 was observed in the four cell lines tested, regardless of the species origin and cell type (epithelial, macrophagic or trophoblastic), confirming the intracellular attenuation of the Rev1 vaccine strain. In addition to the delay in establishing the rBCV, the Rev1 intracellular attenuation might result from a defect in intracellular fitness, as shown by the reduced total multiplication and replication rates of Rev1 when compared to *Bm*16M, supported by the slower growth curve profiles in culture media. Despite this intracellular attenuation, Rev1 could subvert intracellular trafficking and establish the calnexin^+^ compartment and replicate, showing that once the replicative niche is reached and bacterial replication begins, the vaccine replicates in high numbers. This efficient intracellular replication of the Rev1 vaccine is consistent with its high virulence, particularly in the reproductive tract, and its abortifacient effects. This highlights the need to develop safer vaccines that maintain efficacy while minimising adverse outcomes. Achieving this balance is essential to enable wider vaccine use, particularly in endemic regions where mass vaccination campaigns are key to effective brucellosis control.

It is important to note that, regardless of the origin of the cell lines evaluated in this work, whether bovine or ovine, both *B. abortus* and *B. melitensis* strains were able to infect and replicate in high numbers. No evident differences regarding the species preference of these *Brucella* spp. were found, at least to the extent of our investigation. This speaks about the idea that regardless of *B. abortus* being typically found in cattle and *B. melitensis* in sheep (and goats), pathogenesis and animal disease are developed when there are epidemiological opportunities. In fact, *B. melitensis* can establish permanent infections in cattle while, although reports are rare, *B. abortus* can also infect sheep or goats [80]. In other words, species preference is not species-restrictiveness, at least in terms of intracellular multiplication in the studied trophoblasts and macrophages. Overall, the different ruminant-derived target cell lines validated in this work may serve as a more biologically relevant set-up to study the intracellular pathogenesis of *Brucella* spp.

## Supplementary Information

**Additional file 1. List of bacterial strains employed.**

**Additional file 2.** **The constructed ::Tn7GFP-expressing strains did not exert a growth defect with regard to their respective parental strains.** Curve values at each time-point represent the mean ± standard deviation (error bars are represented as the area within the respective values) of an experiment performed in technical triplicates. The experiment was repeated three times with similar results.

**Additional file 3.** **Superplot of the intracellular bacterial burden of *B. abortus* 2308W, *B. melitensis* 16M, *Bm*16MΔ*virB* and Rev1 strains in (A) AH-1 ovine trophoblasts, (B) F3 bovine trophoblasts, (C) BoMac bovine macrophages and (D) HeLa human epithelial cells.** Cells were seeded on crystal coverslips at 1 x 10^4^ cells/well (A) or 2 x 10^4^ cell/well (B-D) and infected at an MOI=100 (A-C) or 1000 (D) of GFP-expressing *Ba*2308W, *Bm*16M, *Bm*16MΔ*virB* and Rev1, and fixed at 2, 24 or 48 hpi and immunostained for LAMP1 or calnexin. Intracellular bacterial burden was determined by IF microscopy by estimation of intracellular bacterial burden based on the surface of fluorescent signal along the Z-stack within 50-infected-cells, all values are represented as a superplot and as mean ± SEM of the individual counts. Statistical comparisons were made by ordinary one-way ANOVA and Sidak’s multiple comparisons post-hoc test (ns = not significant; * *p* < 0.05; ** *p* < 0.01; *** *p* < 0.001; **** *p* < 0.0001.

 **Additional file 4. The Rev1 attenuated vaccine-strain infects F3 bovine trophoblasts, BoMac bovine macrophages and HeLa human epithelial cells at the same level as the *B. melitensis* parental strain (*Bm*16M) but develops an imperfect intracellular trafficking and a delayed intracellular multiplication in LAMP1**^**-**^**/calnexin**^**+**^** BCVs with lower replication values.** (A) Infectivity was determined as the number of infected cells at 2 hpi from 20 screened quadrants (minimum of 140 cells screened) and expressed as mean percentage of infected cells ± SEM, obtained from independent biological triplicates (colour coded: yellow, blue, purple). (B) LAMP1 positivity was determined for each of the BCVs found within each 50 infected cell and expressed as mean percentage of LAMP1^+^-BCVs/infected-cell ± SEM, obtained from independent biological triplicates (colour coded: yellow, blue, purple). (C) Representative pictures of Rev1-infected AH-1 ovine trophoblasts at 48 hpi with LAMP1+ BCVs (left panel) and calnexin^+^ BCVs (right panel) with replicating bacteria. (DAPI: cyan channel; Brucellae: red channel; LAMP1/calnexin: white channel. Scale bars correspond to 5 µm). (D) Bacterial replication levels were determined by CFU-counting and values were expressed as mean log_10_(CFU/mL) ± SD; obtained from technical triplicates and performed two times obtaining similar results. (E) Bacterial replication profiles were determined by IF microscopy by quantification of intracellular bacteria within 50 infected cells and values were classified within the categories of non-replicative (0-9), initiating low replication (10-49), high replication (50-499) and extensive replication (>500 intracellular bacteria); obtained from biological triplicates. Statistical comparisons were made by ordinary one-way ANOVA and Tukey’s (A) or Sidak’s (B) multiple comparisons post-hoc tests, by unpaired t test (C) or by two-way ANOVA and Sidak’s correction (D): ns = not significant; * *p* < 0.05; ** *p* < 0.01; *** *p* < 0.001; **** *p* < 0.0001. Results for the *Bm*16M strain are duplicated from the ones found in Figures 1-3.

**Additional file 5**. **The Rev1 vaccine-strain shows lower intracellular replication rates than the *B. melitensis* (*Bm*16M) wild-type strain.** Bacterial replication levels were determined by CFU-counting at 2, 24 and 48 hpi by cell lysis and plating, and expressed as mean log_10_ (fold-change) ± SD in bacterial burden between 2-24, 24-48 hpi time-periods; obtained from technical triplicates. Statistical comparisons were made by unpaired t-test (ns = not significant; * *p* < 0.05; ** *p* < 0.01; *** *p* < 0.001; **** *p* < 0.0001).

## Data Availability

All data supporting the findings of this study are available within the paper and its Supplementary Information.
