# Supplementary material for: Pathogenesis of Brucella abortus and Brucella melitensis in bovine and ovine-derived trophoblasts and macrophages and impaired intracellular trafficking of the Rev1 vaccine strain
Source: Vet Res. 2026 Jul 7;57:124. doi: 10.1186/s13567-026-01781-3 (PMC13339977; doi:10.1186/s13567-026-01781-3)
Supplement: Supplementary file 1 — Additional file 1. List of bacterial strains employed. [file 13567_2026_1781_MOESM1_ESM.pdf]

Additional file 1. List of bacterial strains employed.

	Characteristics	Source or reference
**Bacterial strains**		
Brucella melitensis		
B. melitensis 16M	*B. melitensis* biovar 1 16M nalidixic acid -resistant spontaneous mutant; virulent.	UNAV collection [26, 27]
*Bm*16M::Tn7GFP	*B. melitensis* biovar 1 16M complemented by chromosomal insertion with a miniTn7 transposon carrying the *gfpmut3* gene; Km^R.	This work
*Bm*16MΔ*virB*	*B. melitensis* biovar 1 16M mutant strain lacking a functional VirB system (non-polar mutant in *virB10*).	Centre d'Immunologie de Marseille-Luminy collection
*Bm*16MΔ*virB*::Tn7GFP	*B. melitensis* biovar 1 16M mutant strain lacking a functional VirB system complemented by chromosomal insertion with a miniTn7 transposon carrying the *gfpmut3* gene; Km^R.	This work
Rev1	*B. melitensis* Rev1 vaccine reference strain.	CITA collection
Rev1::Tn7GFP	Rev1 vaccine-strain complemented by chromosomal insertion with a miniTn7 transposon carrying the *gfpmut3* gene; Km^R	This work
B. abortus		
B. abortus 2308W	*Brucella abortus* 2308 virulent biovar 1, smooth LPS, spontaneous nalidixic acid resistant. It is virulent in mice and has been sequenced and found to differ from 258 and 258A.	UNAV collection [28]
*Ba*2308W::Tn7GFP	*Brucella abortus* 2308W complemented by chromosomal insertion with a miniTn7 transposon carrying the *gfpmut3* gene; Km^R.	This work
Escherichia coli		
S17λpir pUC18R6KT-mini-Tn7-*gfp*-Km	Tp^R $Strp^R$ *recA thi hsdRM*+, lambda pyr phage lysogen RP4::2-Tc::Mu::Km Tn7.	UNAV collection
SM10λpir (pTNS2)	pTNS2 is a helper plasmid encoding the TNsABC+D transposition pathway (Amp^R).	[33]
HB101 (pRK2013)	pRK2013 is a helper plasmid for mobilization of non-self-transmissible plasmids (Km^R).	[33]

[1] Amp, ampicillin; CITA, Centro de Investigación y Tecnología Agroalimentaria de Aragón; DAP, 2,6-diaminopimelic acid; Km, kanamycin; Nal, nalidixic acid; ^{R}, resistant; Strp, streptomycin; Tc, tetracycline; Tp, trimethoprim.
