# Supplementary figures and images for: Pathogenesis of Brucella abortus and Brucella melitensis in bovine and ovine-derived trophoblasts and macrophages and impaired intracellular trafficking of the Rev1 vaccine strain
Source: Vet Res. 2026 Jul 7;57:124. doi: 10.1186/s13567-026-01781-3 (PMC13339977; doi:10.1186/s13567-026-01781-3)

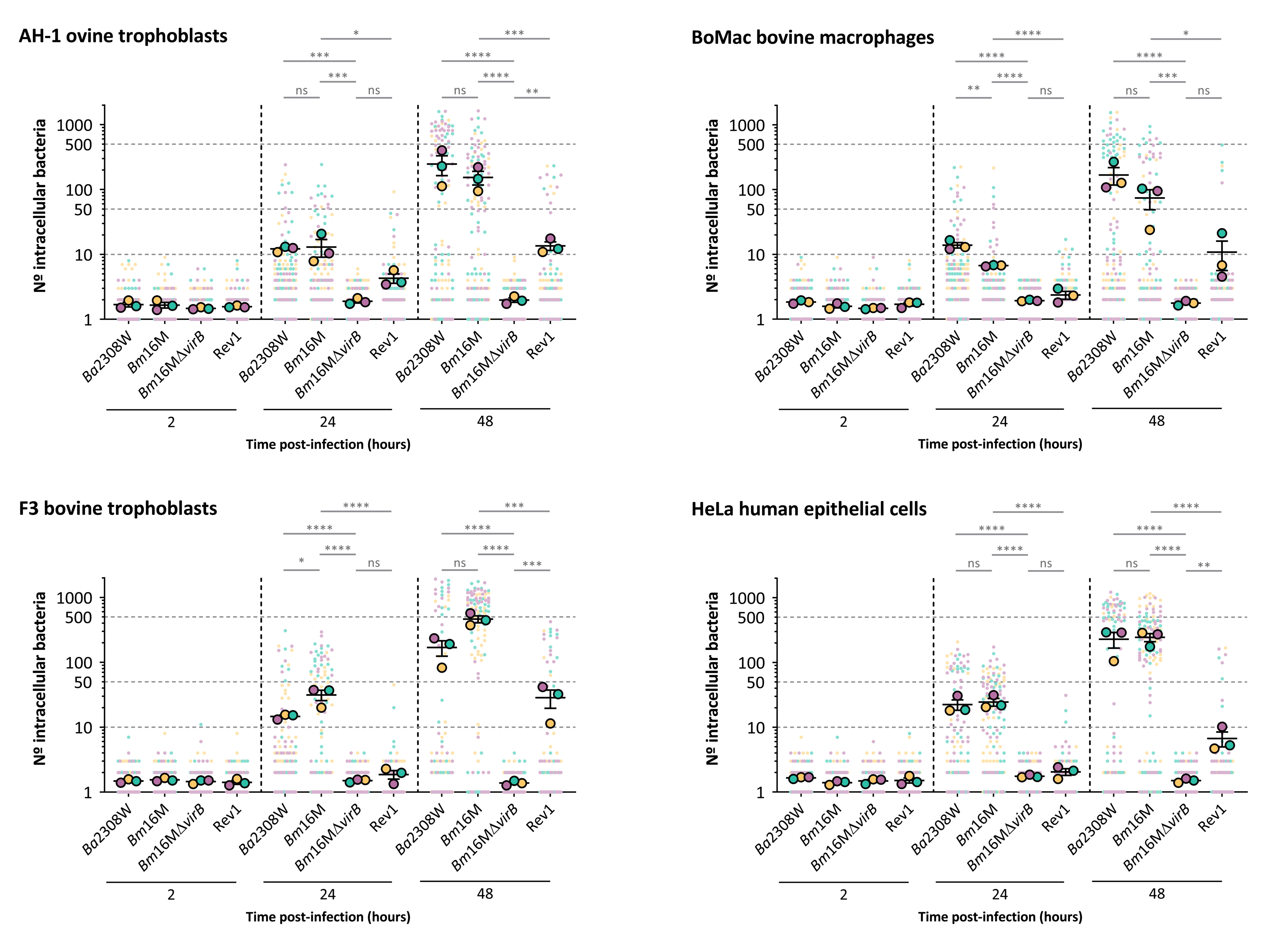

Supplement: Supplementary file 3 — Additional file 3. Superplot of the intracellular bacterial burden of B. abortus 2308W, B. melitensis 16M, Bm16MΔvirB and Rev1 strains in (A) AH-1 ovine trophoblasts, (B) F3 bovine trophoblasts, (C) BoMac bovine macrophages and (D) HeLa human epithelial cells. Cells were seeded on crystal coverslips at 1 x 10^4^ cells/well (A) or 2 x 10^4^ cell/well (B-D) and infected at an MOI=100 (A-C) or 1000 (D) of GFP-expressing Ba2308W, Bm16M, Bm16MΔvirB and Rev1, and fixed at 2, 24 or 48 hpi and immunostained for LAMP1 or calnexin. Intracellular bacterial burden was determined by IF microscopy by estimation of intracellular bacterial burden based on the surface of fluorescent signal along the Z-stack within 50-infected-cells, all values are represented as a superplot and as mean ± SEM of the individual counts. Statistical comparisons were made by ordinary one-way ANOVA and Sidak’s multiple comparisons post-hoc test (ns = not significant; * p < 0.05; ** p < 0.01; *** p < 0.001; **** p < 0.0001. [file 13567_2026_1781_MOESM3_ESM.tiff]

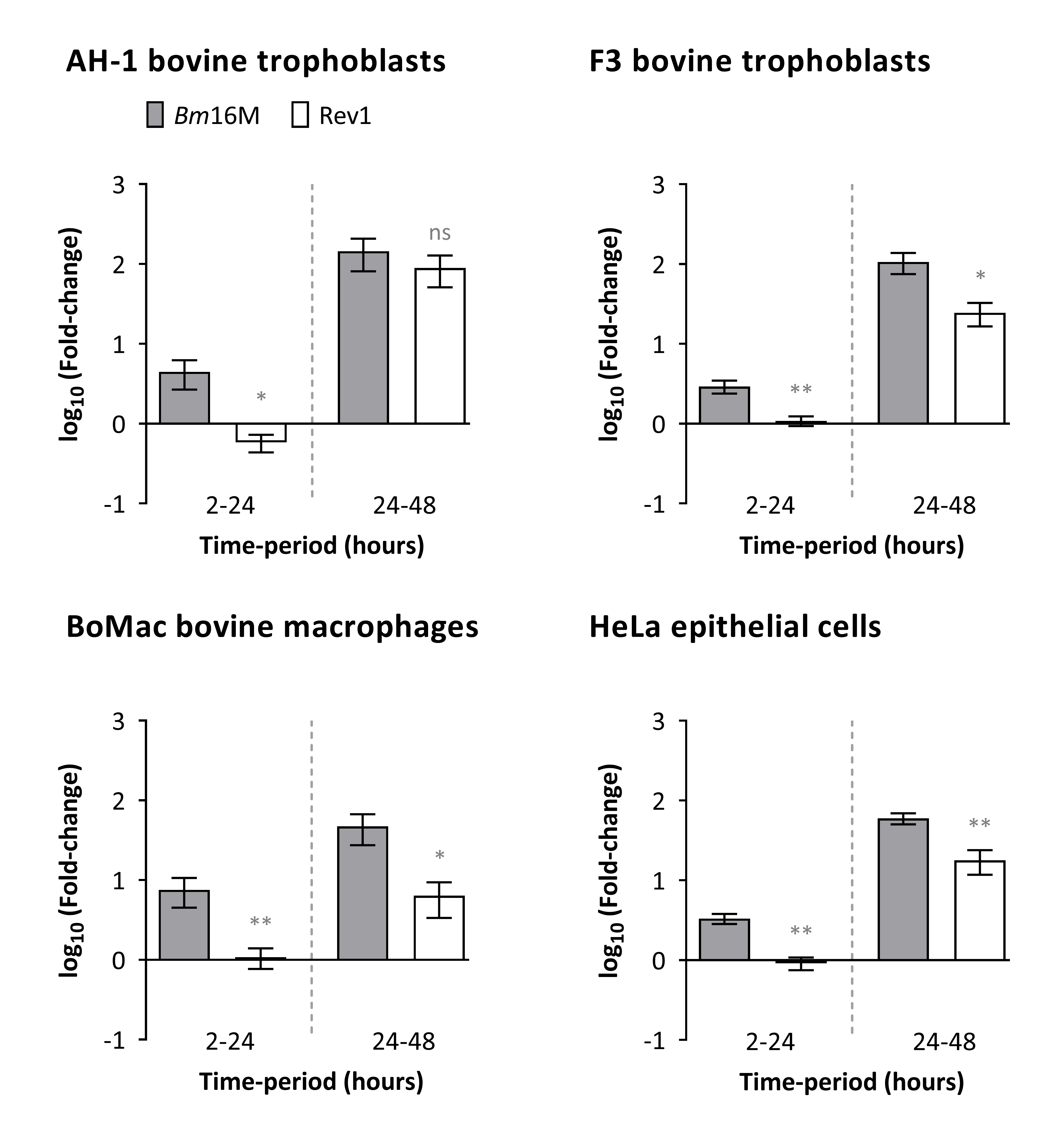

Supplement: Supplementary file 5 — Additional file 5. The Rev1 vaccine-strain shows lower intracellular replication rates than the B. melitensis (Bm16M) wild-type strain. Bacterial replication levels were determined by CFU-counting at 2, 24 and 48 hpi by cell lysis and plating, and expressed as mean log10 (fold-change) ± SD in bacterial burden between 2-24, 24-48 hpi time-periods; obtained from technical triplicates. Statistical comparisons were made by unpaired t-test (ns = not significant; * p < 0.05; ** p < 0.01; *** p < 0.001; **** p < 0.0001). [file 13567_2026_1781_MOESM5_ESM.tif]
